# New York College of Veterinary Surgeons; Commencement

**Published:** 1891-05

**Authors:** Harry D. Gill

**Affiliations:** Secretary


### Veterinary College Notes.

*New York College of Veterinary Surgeons and School of Comparative Medicine; Commencement.*—The 34th annual commencement of the New York College of Veterinary Surgeons and School of Comparative Medicine, was held at Chickering Hall, March 5, 1891. The hall was filled to its utmost capacity by an interested audience. Conterno's 9th Regiment Band furnished music. The President of the College accompanied by the Trustees and Faculty, took the platform, and immediately following them the graduating class filed in and occupied the first four rows of seats; the following three rows being occupied by Juniors who were entitled to certificates.

Dr. Harry D. Gill, Secretary of the Faculty, presented to President Wm. T. White, M. D., the names of those students who had complied with all necessary legal requirements and had passed a satisfactory examination, and he, after duly obligating them, conferred upon them the degree of V. S., as follows :

R. Lindsay Tritton, Boonton, N. J.; John D. Deal, Charleston, Mo.; James Haley, Jr., Hamilton, Scotland; Pleasant H. Browning, Virden, Ill.; Robert P. McDougall, Wilmington, N. C.; Edward Plummer, Jr., M. D. Baltimore, Md.; Wesley F. Strong, Washington, D. C.; Edgar Chambers, New York City; James H. Ferster, New York City; August C. Hassloch, New York City; Robert N. Manney, New York City; Robert J. McNair, New York City; Charles E. Caulfield, New York City; James H. Mullen, New York City; James H. Lawler, New York City; William H. Brooks, Brooklyn, N. Y.; John J. Curran, Brooklyn, N. Y.; Louis J. Meinck, Brooklyn, N. Y.; Charles F. Moadinger, Jr., Brooklyn, N. Y.; Charles E. Anderson, City Island, N. Y.; D. Edgar Smith, Manhasset, N. Y.; James B. Shearer, Pittsford, N. Y.; William J. Doyle, Auburn, N. Y.; Thomas S. Childs, Troy, N. Y.; William Stinson, Chelsea, Mass.; Edwin P. Henderson, Chelsea, Mass.; Charles S. Moore, Plainville Mass.; Monroe B. Miller, Old Line, Pa.; Harry K. Miller, Mannheim, Pa.; William B. Collom, Doylestown, Pa.; E. Michener Massinger, Chalfont, Pa.; Morris M. Downs, West Chester, Pa.; Robert J. Fox, Bryn Mawr, Pa.; Henry S. Christy, Philadelphia, Pa.; Harry F. Albright, Allentown, Pa.; Edward J. Young, Media, Pa.

Prof. C. B. Michener, awarded the following prizes :

Gold medal for best general examination, August C. Hassloch; Silver medal for best Junior examination, W. H. Wilson. The Comstock Prize, a Pocket Case of Instruments, for best anatomical preparation, R. Lindsay Tritton; Jenkin Prize, Set of Standard Veterinary Works, for best written Thesis, C. F. Moadinger, Jr. The Reyling Prize, an Emergency Valise, for the best paper on Materia Medica, Edgar Chambers. The Valedictory address was delivered in a masterly and impressive manner by Dr. Harry F. Albright.

Rev. J. B. English. D. D., M. D., was then introduced to the audience, and his address, sandwiching scientific facts with equine anecdotes, kept the audience in alternate states of profound interest and mirthful demonstration. The floral display embraced many beautiful and unique designs.

The graduating class and invited guests, repaired to Martinelli's for a spread, at which the following toasts were given :

The New York College of Veterinary Surgeons; Prof. H. D. Gill; the Veterinary Profession, Prof. C. B. Michener; the Class of '91, Dr. Jas. Haley; Our Guests, Prof. Carroll; the Medical Profession, Prof. H. M. Biggs; The Press, Dr. C. C. Catenach; Human and Veterinary Medicine, their corelation and their common interest, Prof. Geo. A. Lyons; Dr. J. H. Ferster, Toast Master.

*Class Officers.*—Edgar Chambers, President; John D. Deal; Vice-President; Pleasant H. Browning, Secretary; J. H. Ferster, Treasurer.

*Executive Committee.*—M. B. Miller, Chairman; J. Haley, Secretary; Wm. Stinson, Chas. E. Caulfield, Morris M. Downs.

HARRY D. GILL, *Secretary.*

